# Moth Wing Scales Slightly Increase the Absorbance of Bat Echolocation Calls

**DOI:** 10.1371/journal.pone.0027190

**Published:** 2011-11-09

**Authors:** Jinyao Zeng, Ning Xiang, Lei Jiang, Gareth Jones, Yongmei Zheng, Bingwan Liu, Shuyi Zhang

**Affiliations:** 1 Institute of Molecular Ecology and Evolution, Institutes for Advanced Interdisciplinary Research, East China Normal University, Shanghai, People's Republic of China; 2 School of Architecture, Rensselaer Polytechnic Institute, Troy, New York, United States of America; 3 National Center for Nanoscience and Technology, Beijing, People's Republic of China; 4 School of Biological Sciences, University of Bristol, Bristol, United Kingdom; 5 College of Wildlife Resources, Northeast Forestry University, Harbin, People's Republic of China; University of California Merced, United States of America

## Abstract

Coevolutionary arms races between predators and prey can lead to a diverse range of foraging and defense strategies, such as countermeasures between nocturnal insects and echolocating bats. Here, we show how the fine structure of wing scales may help moths by slightly increasing sound absorbance at frequencies typically used in bat echolocation. Using four widespread species of moths and butterflies, we found that moth scales are composed of honeycomb-like hollows similar to sound-absorbing material, but these were absent from butterfly scales. Micro-reverberation chamber experiments revealed that moth wings were more absorbent at the frequencies emitted by many echolocating bats (40–60 kHz) than butterfly wings. Furthermore, moth wings lost absorbance at these frequencies when scales were removed, which suggests that some moths have evolved stealth tactics to reduce their conspicuousness to echolocating bats. Although the benefits to moths are relatively small in terms of reducing their target strengths, scales may nonetheless confer survival advantages by reducing the detection distances of moths by bats by 5–6%.

## Introduction

Moths and butterflies comprise the order Lepidoptera, which is the second largest insect group in terms of species richness. The fossil record indicates that moths have existed for around 140 million years and butterflies nearly 70 million years [1], [2]. Bats evolved later than moths, perhaps 70 million years ago [3], [4] and are the main predators of nocturnal aerial insects. Moths have evolved a suite of defenses to reduce their chances of being eaten by bats, including the possession of tympanic organs that are sensitive to ultrasound in many taxa, the ability to adjust flight paths after detecting bats and even the use of ultrasound to deter or jam the echolocation of approaching bats [5], [6], [7].

The wings of moths and butterflies are made of thin layers of chitin, covered with numerous flat scales that are usually less than 0.25 mm wide [8]. Some of these scales contain rich chemical pigments that give rise to bright colours, others contain millions of microscopic ridges and bumps that cause light to bounce off in different directions and create brilliant and iridescent structural colours [9]. The colours and patterns of moth and butterfly wings elaborated by scales serve several functions: (*i*) mate attraction [10] or mate choice [11]; (*ii*) warning predators that the insect is toxic [12]; (*iii*) camouflage or mimicry that may assist in protection against predators [13], (*iv*) absorbing or reflecting sunlight as tiny solar heat collectors and aiding in temperature regulation in order to warm insects up for flight or even by acting as reflectors to protect insects from overheating as feathers may do in birds [14], [15], (*v*) offering protection against spiders by shedding and sticking to adhesive in webs [16], (*vi*) aiding in sound production or acting as a sound resonator [17]. We hypothesize that a potentially vital function of moth scales is to absorb the ultrasonic echolocation calls emitted by bats; this aspect of moth biology has only been investigated crudely in previous studies [18], [19]. Here, we use a novel method for measuring ultrasound absorption from small biological samples and demonstrate that some moths have evolved additional stealth tactics to reduce their conspicuousness to echolocating bats by their wing scales absorbing ultrasound.

## Results

**Figure 1:**
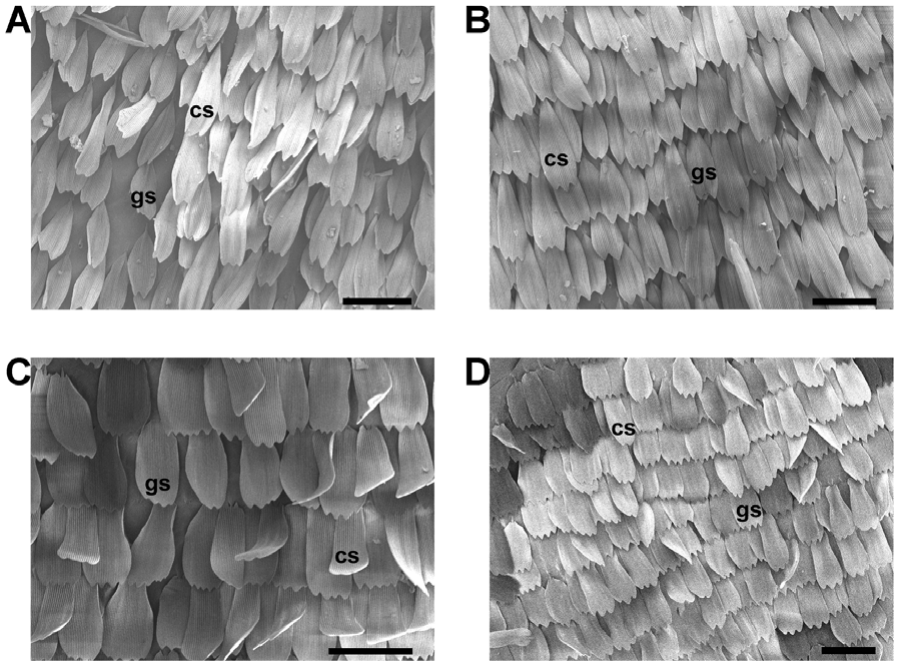
SEM images of a tiny section of the hind wing. Showing the arrangement of ground scales (gs) and cover scales (cs) on the wing. (*A*) The Tiger Moth (*Spilosoma niveus*). (*B*) The Clouded Buff (*Rhyparioides amurensis*). (*C*) The Indian Cabbage White Butterfly (*Pieris canidia*). (*D*) The Asian Pale Clouded Yellow (*Colias erate*). Scale bar, 100 µm.

We compared the structural characteristics of scales in two moth and two butterfly species, and measured their absorptivity of different ultrasonic frequencies. We chose the Tiger Moth (*Spilosoma niveus*) and Clouded Buff (*Rhyparioides amurensis*) moths (Arctiidae), and the Indian Cabbage White (*Pieris canidia*) and Asian Pale Clouded Yellow (*Colias erate*) butterflies (Pieridae) as model species because these are among the most common moth and butterfly species in China. Two distinct layers—ground and cover scales—can be observed in the wings of these lepidopterans (Figure 1). Approximately 100 million scales are present on each wing and the scale dimensions are shown in Table 1.

**Table 1:** Characteristics of cover scales and ground scales in the moths and butterflies.

Species		Cover scales		Ground scales	
		Length (**µ**m)	Width (**µ**m)	Length (**µ**m)	Width (**µ**m)
Moths	*Spilosoma niveus*	174.38±1.72 (*n* = 70)	44.17±0.44 (*n* = 70)	96.28±1.75 (*n* = 105)	42.56±0.44 (*n* = 105)
	*Rhyparioides amurensis*	220.58±3.42 (*n* = 50)	44.81±1.01 (*n* = 50)	135.44±1.67 (*n* = 110)	48.97±0.7 (*n* = 110)
Butterflies	*Pieris canidia*	129.56±1.57 (*n* = 20)	33.17±1.32 (*n* = 20)	101.86±1.18 (*n* = 90)	54.76±0.54 (*n* = 90)
	*Colias erate*	125.82±1.06 (*n* = 30)	39.47±0.45 (*n* = 30)	98.38±0.73 (*n* = 120)	51.9±0.39 (*n* = 120)

Values are expressed as mean ± SEM.

**Figure 2:**
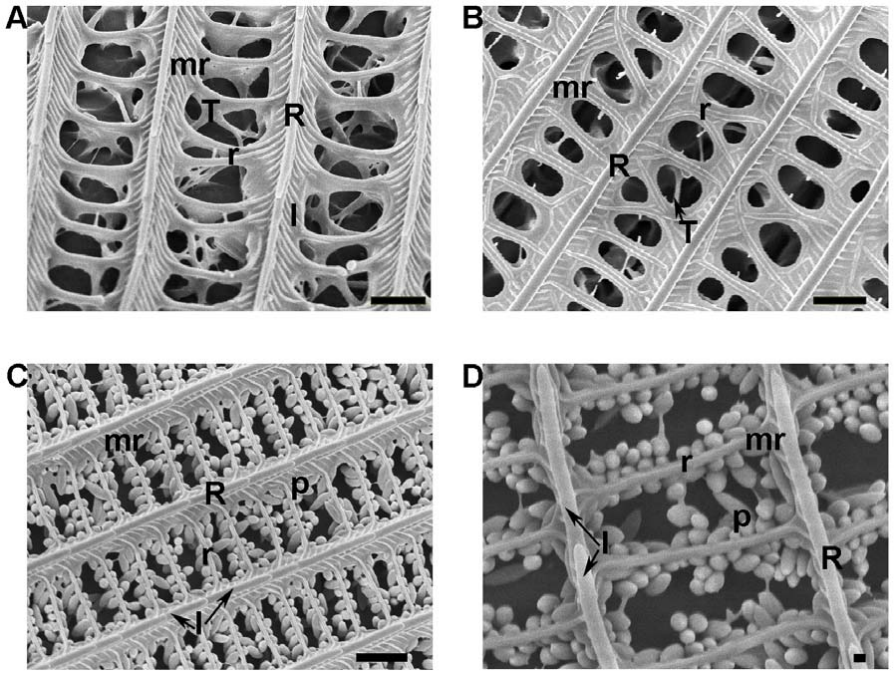
SEM images of a scale fragment. Showing the complicated upper lamina and the interior of a scale. Ridges (R), lamellae (l), microribs (mr) and crossribs (r) are indicated distinctly. (*A* and *B*) Multiform trabeculae (T) connect with each other and form various mesh between the upper and bottom laminae within the scale of *Spilosoma niveus* (*A*) and *Rhyparioides amurensis* (*B*). Scale bar, 1 µm. (*C* and *D*) These meshworks all run into one another within the scale. Ridges and microribs form many close and regular windows, and pigment granules (p) could be seen in *Pieris canidia* (*C*) and *Colias erate* (*D*) scales, although trabeculae were not apparent. Scale bars, 1 µm (*C*), 100 nm (*D*).

As described for other lepidopterans [8], the scales of both insects display similar basic structural components: longitudinal ridges, overlapping lamellae, microribs, transverse crossribs and pillar-like trabeculae (Figure 2). In the scales of the moths that we examined, the regions between the ridges and crossribs are hollow, as a consequence of their multilayer microstructure. Hollows connect with each other and form a meshwork. However, no equivalent hollows were observed in the butterfly scales, in which some pigment granules were attached to the ridges (Figure 2).

**Figure 3:**
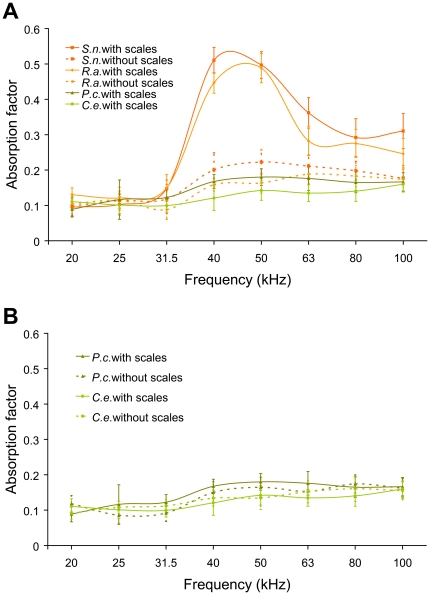
Absorption factor of wings with and without scales of moths and butterflies from 20 kHz to 100 kHz. *Spilosoma niveus* (*S.n.*), *Rhyparioides amurensis* (*R.a.*), *Pieris canidia* (*P.c.*) and *Colias erate* (*C.e.*). (*A*) Moths with scales differed significantly in absorption factors compared with moths without scales, and from butterflies with and without scales (*P*<0.001). Absorption factors of moths without scales did not differ from those of butterflies either with or without scales (*P*<0.05). (*B*) The absorption factors of butterfly wings with or without scales did not differ significantly (*P*>0.05). Experiments were performed in triplicate, and the SEM is indicated by the error bars, and *n* = 15 for all samples.

**Figure 4:**
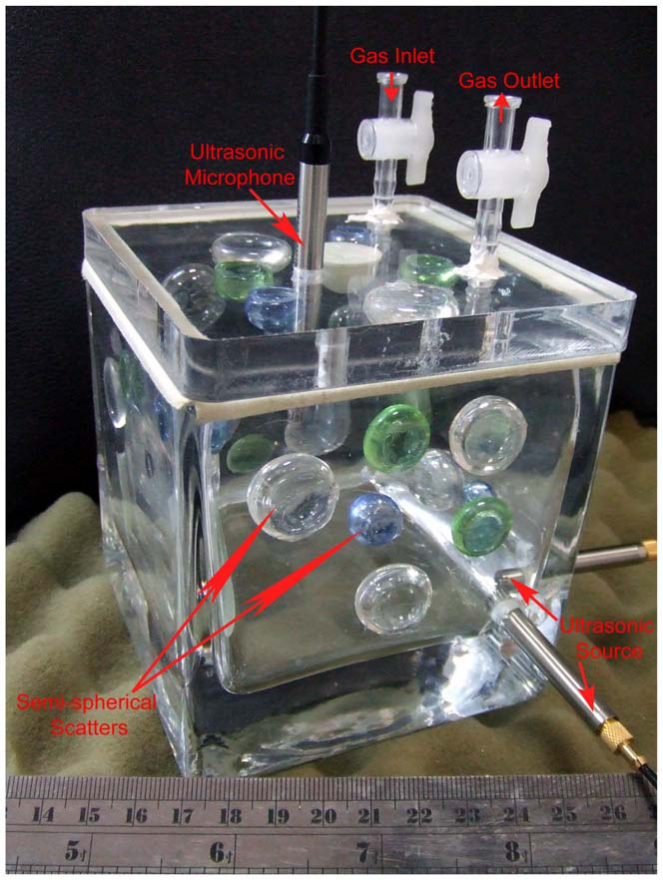
Reverberation chamber used for the ultrasonic absorption measurements. The ultrasonic source is deployed in a chamber corner, and the ultrasonic microphone can be moved from the lid up-and-down to warrant spatial averages. Many semi-spherical scatters are also deployed on the interior surfaces to achieve a diffuse sound field within the frequency range of interest. Medium exchange by nitrogen is carried out by one of the gas inlet/outlets.

The absorptivity of ultrasound by moth and butterfly wing fragments (10 cm^2^: *n* = 15 samples for each treatment) was measured over the frequency range 20–100 kHz (Figure 3), using a reverberation chamber (Figure 4). The random-incident absorption factors of the moth wings with and without scales were also measured. The absorption factors of the two moths were considerably higher than that of two butterflies between 40–60 kHz. When the scales were removed, the peaks of the absorption factors of the moths declined markedly (Figure 3A). The absorption factors among the butterflies' wings with and without scales were not different (Figure 3B). A repeated measures analysis of variance (with ultrasound frequency as within-subject factors and presence/absence of scales the moths/butterflies as between - subject factors) showed a significant effect of preparation type (moths or butterflies with or without scales) (*F*_3,959_ = 76.17, *p*<0.001) and frequency (*F*_7,959_ = 29.53, *p*<0.001) on absorption factor. Moreover, there was a significant group×frequency interaction (*F*_21,959_ = 8.87, *p*<0.01) showing that different species had different absorption factors at different frequencies. Bonferroni post-hoc comparisons showed that moths with scales differed significantly in absorption factors compared with moths without scales (*p*<0.01) and from butterflies with (*p*<0.01) and without (*p*<0.01) scales. However, when moth scales were removed, the absorption factors did not differ from those of butterflies either with (*p* = 0.45) or without (*p* = 0.26) scales. The absorption factors of butterfly wings with or without scales did not differ significantly (*p* = 1.00). These results show that the moth scales increase the absorption of ultrasound significantly compared with butterfly scales and that this absorptive advantage is lost when the scales are removed only for moths.

## Discussion

It is unlikely that moth scales function as heat reflectors to protect moths from overheating most moths are nocturnal, and temperatures at night are generally not as high as during the day. That the scales function as solar panels for warming can also be rejected for similar reasons, though scales may assist in maintaining relatively high body temperatures at night. Scales also confer colour to moths, assisting with protection against visual predators by aposematism or camouflage. Scales may also protect moths from becoming ensnared in spider webs, and these potential benefits are not mutually exclusive from any acoustic benefits of scales.

Preliminary indications of a possible anechoic role for moth scales have nearly always come from studies on bats' perception of moths as prey. It has been firmly established that bats determine species-specific features of moth prey depending on their shape, size and depth-structure [20], [21], [22]. The observation that echo amplitudes increase by 1 to 2 decibels by removal of moth scales was first measured by Roeder, although the effect was insignificant compared with that produced by changes in moth wing positions [18]. These estimates correspond closely with our values – the difference between target strengths of scaled and unscaled moths at 40 kHz (where the difference is greatest) is only estimated to be about 2 dB (A. T. Catherall pers. comm.). Since Roeder's measurements, more has become known of the intensity of bat echolocation calls and the hearing thresholds of bats, and so it is possible to model the advantages conferred by scales in reducing detection distances of moths by bats.

Target strengths for moths of the size studied here are typically around −15 dB [23]. We used the models of Goerlitz et al. [24] to calculate changes in detection distance of moths by bats. Assuming a source level of 125 dB SPL at 10 cm, 17°C and 80% relative humidity, the model predicts detection distances of 11.24 m (unscaled) and 10. 75 m (scaled) assuming a bat hearing threshold of 0 dB SPL, and 6.75 m and 6.36 m respectively with a hearing threshold of 20 dB SPL. These predictions suggest that scales might confer a 5–6% difference in detection distance at best, which is small but potentially of evolutionary significance to enhancing moth survival prospects.

In addition, the sound absorbing role of wing scales was also suggested by observations on spectral modifications in echoes, in which the relative height of the side lobes was changed in the cross-correlation functions, and notches appeared in power spectra and in spectrograms [19]. Yet relatively little is known about the detailed mechanisms by which wing scales might reduce echo strengths. In this comparative study on wing scale absorption, we confirmed that moth scales absorb some frequencies of sound, and that the optimal absorbing frequency range is 40–60 kHz.

The mechanisms responsible for sound absorption of wing scales are currently unknown. Numerous works have been devoted to colour effects in iridescent scales in Lepidoptera, especially in butterflies [8], [9], [25]. Several other functions of scales as the constituents of auditory, scent or sound-producing organs have also been reported [17], [26], [27]. Scale patterns obtained in this study showed that the scales overlap in butterflies in a more ordered manner (like roof-tiles) than in moths. Butterfly scales are also more uniform in structure. Perhaps, the more scattered arrangement of moth scales creates larger interstitial air space hollows, which may act as a porous absorber so that sound propagation occurs in a network of interconnected pores though viscous effects causing acoustic energy to be dissipated as heat [28]. Furthermore, the ultrastructure of moth scales is similar to a thin panel perforated with a large number of microscopic pores together with an air space behind it, which probably acts as a microperforated panel absorber [29]. Such a structure resembles the acoustically absorptive material used by humans in buildings, however, the sound absorbers of the moth scales may be more effective than some man-made absorbers, because there is an impedance-matching structure between the air and the perforated panel, which consists of wedges similar to those used in an anechoic chamber. These wedges could further reduce the reflection of sound although the perforated panel will have initially absorbed most of the sound. We therefore suggest that there is probably an integrative action with both a porous absorber and a perforated panel absorber that accounts for the sound absorption in moth wings.

Most echolocating bats emit calls that contain most energy between 20–60 kHz [22], [30]. In China, the commonest aerial feeding bat, *Pipistrellus abramus*, echolocates with most energy at 44–52 kHz [31]. This frequency band accords well with that best absorbed by the moth scales. We believe this might be the result of selective pressure from the major predators of moths–echolocating bats. The structure of the sound absorbers on the moth wings would reduce the chance of the moth being detected by bats. Future experiments will reveal how widespread the absorbance of ultrasound is among moth species, and whether absorbance is related to the range of echolocation calls emitted by sympatric bat species. Another particularly interesting area for future research might be to confirm the mechanisms of absorbance. Many moths have already evolved ears to detect echolocating bats, and undertake evasive manoeuvres to escape from them [7]. Stealth would therefore be an extra defense used by moths in avoiding predation by echolocating bats in a way similar to the use of absorbent materials used to make military aircraft less detectable by radar, and submarines less detectable by sonar [32].

In conclusion, some moths carry scales that have hollows between ridges and crossribs in their structure, and these were not recorded in the butterfly species studied. Moth wing fragments reflected weaker echoes than did butterfly wing fragments of equivalent size, and the removal of scales from moth wings decreased their absorbance of ultrasound, while a similar effect did not occur in butterflies. We suggest that moth scales may confer a marginal acoustic advantage to moths by decreasing the distance that bats can detect the moths by 5–6% in a best-case scenario. Further studies on how widespread the differences in scale structure that we observed are (as we only investigated moths and butterflies from two families), and whether the removal of scales increases the predation risk to moths will be revealing.

## Materials and Methods

### (a) Study species

Tiger Moths *Spilosoma niveus* (Ménétriés, 1859) and the Clouded Buff *Rhyparioides amurensis* (Bremer, 1861), the Indian Cabbage White Butterflies *Pieris canidia* (Linnaeus, 1768) and the Asian Pale Clouded Yellow *Colias erate* (Esper, [1805]) were captured in rural areas of Beijing and Shanghai. They are all widespread species and usually considered as agricultural pests [33].

### (b) SEM

We determined the shape and surface texture of the scales by obtaining scanning electron microscope (SEM) images from small sections of a wing. Samples were coated with 5 nm of gold to provide a conducting surface, and were examined with a Hitachi S-3200N electron microscope.

### (c) Determination of sound absorption

A micro-reverberation chamber with a known volume was used to measure sound absorptivity of moth and butterfly wings. The steady-state diffuse ultrasonic energy decays at a certain rate (so-called reverberation time) inside the chamber when switching off the ultrasonic excitations. After introducing the moth or butterfly wings, the reverberation time changes with respect to those of the empty chamber. After moth wings were measured, the scales were carefully removed by using the liquid polyvinyl alcohol [25], and then the wings without scales were measured once again under the same conditions. The difference in measured reverberation times caused by specimens of known size is used to determine the absorption factors. 12 microphone positions were selected in each measurement to achieve spatial averaging of measured reverberation times. 15 individuals of each species were examined. The data were analyzed using repeated measures ANOVA followed by post-hoc analyses with Bonferroni tests (SPSS 11.0).

Broad-band measurement techniques for experimental determination of ultrasonic reflection and absorption within the frequency range between 20 kHz and 100 kHz have not yet been documented either in major acoustics or in the biology literature. In architectural acoustics sound absorbing materials are often determined in a reverberation chamber of known volume. The interior of the chamber features highly reflective surfaces. The time for the sound energy to decay from its initial steady-state level by 60 dB (one millionth of the initial sound pressure value) is termed reverberation time. If a diffuse sound field inside the chamber can be created, the empty-chamber reverberation time *T*_0_(*f*) in seconds as function of frequency is determined. Introducing a material under test with a known surface area *S*_m_ in m^2^ into the chamber, the new reverberation times, changed to *T*_1_(*f*) in seconds, are measured. The absorption factors of the material under test *α*(*f*) as function of frequency can then be determined using the Sabine equation [34], [35]

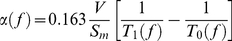
(1)where *V* is the chamber volume in m^3^. The chamber measurement method described above assumes a diffuse sound field inside the chamber, the sound energy impinging upon the material surface under test can be considered randomly from all possible directions, and the background absorption is assumed to be low.

### (d) Micro-reverberation chamber tests

In determining sound absorption of moth and butterfly wings using Eq. (1), this work uses a micro-reverberation chamber with a volume *V* = 504 cm^3^ (Figure 4) and a wing size is in order of *S_m_* = 10 cm^2^. Such a wing size is appropriate for the current investigation on moth wings and was attained by attaching 4 pieces of wing together. The air in the ultrasonic frequency range between 20–100 kHz becomes noticeably absorbent. To compensate the absorbent medium in the reverberation chamber in keeping the background (empty-state) absorption as low as possible, nitrogen is used to replace air in the chamber [36]. During the medium-exchange, once the oxygen content is reduced to 3% (97% nitrogen) in the chamber the measurement can be carried out [36]. A broad-band ultrasonic transducer covering the frequency range between 18 kHz and 100 kHz is deployed inside the chamber near one bottom-corner, while an ultrasonic microphone can be moved up-and-down for measuring the chamber impulse responses at different locations. A broad-band pseudo-random signal is periodically sent to the ultrasonic source at an update-rate of 250 kHz so as to cover the frequency up to 110 kHz, a fast cross-correlation is applied between the excitation signal and the received chamber response measured by the ultrasonic microphone [37], to obtain chamber impulse responses. Using this method, high signal-to-noise ratios (over 45 dB) can be achieved over the frequency range between 20 kHz and 100 kHz. The steady-state ultrasound energy decay is derived from the chamber impulse response by Schroeder integration [38]. A model-based Bayesian energy decay analysis [39] is used to determine the reverberation times. Because the background absorption and the diffusion of the sound field might be chamber-specific, the absorption quantities *α*(*f*) determined using Eq. (1) are termed in this work absorption factors, and these are sufficient to compare the relative absorptivity between moth and butterfly wings under the same acoustic conditions.

## References

[1] Culver SJ, Rawson PF (2000). Biotic response to global change: the last 145 million years.

[2] Vane-Wright D (2004). Butterflies at that awkward age.. Nature.

[3] Teeling EC, Springer MS, Madsen O, Bates P, O'Brien SJ (2005). A molecular phylogeny for bats illuminates biogeography and the fossil record.. Science.

[4] Veselka N, McErlain DD, Holdsworth DW, Eger JL, Chhem RK (2010). A bony connection signals laryngeal echolocation in bats.. Nature.

[5] Fullard JH, Hoy RR, Popper AN, Fay RR (1998). The sensory coevolution of moths and bats.. Comparative hearing: insects.

[6] Miller L, Surlykke A (2001). How some insects detect and avoid being eaten by bats: tactics and countertactics of prey and predator.. Bioscience.

[7] Jones G, Rydell J, Kunz TH (2003). Attack and defense: interactions between echolocating bats and their insect prey.. Bat Ecology.

[8] Ghiradella H (1991). Light and color on the wing: structural colors in butterflies and moths.. Appl Opt.

[9] Vukusic P, Sambles JR (2003). Photonic structures in biology.. Nature.

[10] Burghardt F, Knüttel H, Becker M, Fiedler K (2000). Flavonoid wing pigments increase attractiveness of female common blue (*Polyommatus icarus*) butterflies to mate-searching males.. Naturwissenschaften.

[11] Brunton CFA, Majerus MEN (1995). Ultraviolet colours in butterflies: intra-or inter-specific communication?. Proc R Soc B.

[12] Mallet J, Singer MC (1987). Individual selection, kin selection, and the shifting balance in the evolution of warning colours: the evidence from butterflies.. Biol J Linn Soc.

[13] Brakefield PM, Liebert TG (2000). Evolutionary dynamics of declining melanism in the peppered moth in the Netherlands.. Proc R Soc B.

[14] Kingsolver JG, Watt WB (1983). Thermoregulatory strategies in Colias butterflies: thermal stress and the limits to adaptation in temporally varying environments.. Am Nat.

[15] Srinivasarao M (1999). Nano-optics in the biological world: beetles, butterflies, birds, and moths.. Chem Rev.

[16] Eisner T, Alsop R, Ettershank G (1964). Adhesiveness of spider silk.. Science.

[17] Nakano R, Skals N, Takanashi T, Surlykke A, Koike T (2008). Moths produce extremely quiet ultrasonic courtship songs by rubbing specialized scales.. Proc Natl Acad Sci USA.

[18] Roeder KD (1963). Echoes of ultrasonic pulses from flying moths.. Biol Bull.

[19] Moss CF, Zagaeski M (1994). Acoustic information available to bats using frequency-modulated sounds for the perception of insect prey.. J Acoust Soc Am.

[20] Simmons JA, Chen L (1989). The acoustic basis for target discrimination by FM echolocating bats.. J Acoust Soc Am.

[21] Simmons JA, Moss CF, Ferragamo M (1990). Convergence of temporal and spectral information into acoustic images of complex sonar targets perceived by the echolocating bat, *Eptesicus fuscus*.. J Comp Physiol A.

[22] Waters DA (2003). Bats and moths: what is there left to learn?. Physiol Entomol.

[23] Surlykke A, Filskov M, Fullard JH, Forrest E (1999). Auditory relationships to size in noctuid moths: bigger is better.. Naturwissenschaften.

[24] Goerlitz HR, ter Hofstede HM, Zeale MRK, Jones G, Holderied MW (2010). An aerial-hawking bat uses stealth echolocation to counter moth hearing.. Curr Biol.

[25] Yoshioka S, Kinoshita S (2006). Structural or pigmentary? Origin of the distinctive white stripe on the blue wing of a Morpho butterfly.. Proc R Soc B.

[26] Göpfert MC, Surlykke A, Wasserthal LT (2002). Tympanal and atympanal ‘mouth–ears’ in hawkmoths (Sphingidae).. Proc R Soc B.

[27] Costanzo K, Monteiro A (2007). The use of chemical and visual cues in female choice in the butterfly *Bicyclus anynana*.. Proc R Soc B.

[28] Cox TJ, D'Antonio P (2009). Acoustic absorbers and diffusers: theory, design and application.

[29] Maa DY (1975). Theory and design of micro-perforated-panel soundabsorbing constructions.. Sci Sin.

[30] Fullard JH (1982). Echolocation assemblages and their effects on moth auditory systems.. Can J Zool.

[31] Luo F, Ma J, Li A, Wu FJ, Chen QC (2007). Echolocation calls and neurophysiological correlations with auditory response properties in the inferior colliculus of *Pipistrellus abramus*(Microchiroptera: Vespertilionidae).. Zool Stud.

[32] Denny M (2007). Blip, ping & buzz: making sense of radar and sonar.

[33] Wang P, Wang L, Fang C, Bai J, Zhu H (1981). Iconocraphia Heterocerorum Sinicorum.

[34] Sabine WC (1922). Collected papers on acoustics.

[35] Blauert J, Xiang N (2008). Acoustics for engineers: Troy lectures.

[36] Xiang N, Blauert J (1993). Binaural scale modelling for auralisation and prediction of acoustics in auditoria.. Appl Acoust.

[37] Xiang N, Schroeder MR (2003). Reciprocal maximum-length sequence pairs for acoustical dual source measurements.. J Acoust Soc Am.

[38] Schroeder MR (1965). New method of measuring reverberation time.. J Acoust Soc Am.

[39] Xiang N, Goggans PM (2001). Evaluation of decay times in coupled spaces: Bayesian parameter estimation.. J Acoust Soc Am.

